# Antibiotic and Heavy Metal Resistance in Marine Bacteria from Terra Nova Bay (Ross Sea): Insights from Wild Fish and Environmental Samples

**DOI:** 10.3390/ani16010051

**Published:** 2025-12-24

**Authors:** Enrico Gugliandolo, Bilal Mghili, Francesca Fabrizi, Kannan Gunasekaran, Francesco Smedile, Francesca Inferrera, Sabrina Natale, Teresa Romeo, Erika Arcadi, Syed Sikandar Habib, Maurizio Azzaro, Teresa Bottari, Monique Mancuso

**Affiliations:** 1 Department of Veterinary Science, University of Messina, 98168 Messina, Italy; enrico.gugliandolo@unime.it; 2 LESCB, URL-CNRST N°18, Faculty of Sciences, Abdelmalek Essaadi University, Tetouan 93000, Morocco; b.mghili@uae.ac.ma; 3 Institute for Marine Biological Resources and Biotechnology (IRBIM), National Research Council (CNR), 98122 Messina, Italy; francescafabrizi@cnr.it (F.F.); monique.mancuso@cnr.it (M.M.); 4 Scuola Universitaria Superiore IUSS Pavia, 27100 Pavia, Italy; 5 Department of Marine Science, Faculty of Science, Chulalongkorn University, Bangkok 10330, Thailand; bk.guna18@gmail.com; 6 Institute of Polar Sciences (ISP), National Research Council (CNR), 98122 Messina, Italy; francesco.smedile@cnr.it (F.S.); maurizio.azzaro@cnr.it (M.A.); 7 Department of Chemical, Biological, Pharmaceutical and Environmental Sciences, University of Messina, 98166 Messina, Italy; francesca.inferrera@studenti.unime.it (F.I.); sabrina.natale@unime.it (S.N.); 8 Stazione Zoologica “Anton Dohrn”, Sicily Marine Centre, 98167 Messina, Italy; teresa.romeo@szn.it (T.R.); erika.arcadi@szn.it (E.A.); 9 Stazione Zoologica Anton Dohrn, Sicily Marine Centre, Department of Biology and Evolution of Marine Organisms (BEOM), Via Dei Mille 46, 98057 Milazzo, Italy; 10 National Institute for Environmental Protection and Research, 98057 Milazzo, Italy; 11 National Biodiversity Future Center (NBFC), 90133 Palermo, Italy; 12 Department of Zoology, University of Sargodha, Sargodha Punjab 40100, Pakistan; sikandarzoo00@yahoo.com

**Keywords:** antibiotic susceptibility, heavy metal tolerance, antibiotic resistance genes, *Trematomus bernacchii*, seawater, sediment, Antarctica

## Abstract

This study examines the occurrence of bacteria resistant to antibiotics and heavy metals in Terra Nova Bay, a coastal area of the Ross Sea in Antarctica that is increasingly recognised as vulnerable to human influence. During the 37th Italian Antarctic Expedition (2021–2022), researchers collected seawater, sediment, and fish samples from the notothenioid species *Trematomus bernacchii* to evaluate microbial resistance in an environment once considered largely pristine. Fifty heterotrophic bacterial isolates were obtained and tested against twenty-eight antibiotics, revealing a notable presence of multidrug resistance. These multidrug-resistant isolates were then assessed for their tolerance to eight heavy metal salts to understand whether resistance traits extended beyond antimicrobials. Twelve isolates showing resistance to both antibiotics and metals were selected for further genetic screening, targeting key resistance genes linked to tetracycline, vancomycin, sulphonamides, and other antimicrobial classes. The detection of multiple resistance genes in genera such as *Pseudomonas*, *Pseudoalteromonas*, and *Psychrobacter* indicates that both natural selective pressures and local, human-related contamination may be shaping resistance patterns in this region. Overall, the study demonstrates that even remote Antarctic marine ecosystems can host bacteria with complex resistance profiles. While these ecosystems are largely isolated, human activities such as scientific research, tourism, and the introduction of pollutants may contribute to the dissemination of antibiotic resistance genes, raising important ecological and potential public health considerations regarding the spread of resistance in polar environments.

## Simple Summary

This study investigated bacteria resistant to antibiotics and heavy metals in Terra Nova Bay, Antarctica. During the 37th Italian Antarctic Expedition (2021–2022), researchers collected seawater, sediment and fish samples and isolated 50 bacterial strains. Many of these showed resistance to multiple antibiotics and heavy metals. Twelve particularly resistant isolates also carried specific resistance genes. The findings suggest that both natural selective pressures and local human activities contribute to the spread of these resistances, highlighting potential ecological and public health concerns even in remote Antarctic environments.

## 1. Introduction

Antarctica is a remote, extreme environment, considered for a long time as pristine due to its largely untouched landscapes and minimal human presence [1]. However, global and local sources, such as tourism and the establishment of permanent scientific base, caused significant disturbances and led to the introduction of non-native elements into Antarctic waters [2,3]. Antarctica hosts more than 100 active scientific research stations and seasonal field camps [4]. Several studies have reported localized contamination in terrestrial and marine environments in the vicinity of both active and decommissioned stations [5,6,7,8,9,10,11,12]. These stations create discrete gradients of human activity that can be used as natural laboratories to investigate the influence of anthropogenic inputs on microbial communities, including the occurrence of antibiotic-resistant bacteria [1]. Wastewater treatment systems, present in several Antarctic research stations, represent potential localized sources of pharmaceutical residues and resistant microorganisms, which may influence microbial assemblages in nearby waters and sediments. Consequently, some Antarctic coastal areas exhibit spatially restricted gradients of anthropogenic pressure that allow investigation of how combined environmental and human-related stressors may affect microbial resistance patterns. Resistance traits in Antarctic microorganisms may reflect long-standing natural selective pressures [13,14], such as exposure to naturally occurring metals, as well as exposure to anthropogenic contaminants. Heavy metals including Pb, Cd, Ni, Co, Cu, Hg, Zn, and Cr have been detected in Antarctic environmental matrices and have been associated with co-selection mechanisms linked to antimicrobial resistance [10,15,16,17,18,19,20,21,22]. In addition, microplastics and trace pharmaceutical residues have been shown to provide surfaces for microbial colonization and to act as potential carriers of resistance determinants at local scales [11,23,24,25,26,27]. For these reasons, research efforts have focused on characterizing localized contamination by hydrocarbons, antibiotics, and heavy metals, together with the possible introduction of non-native bacteria carrying resistance genes associated with these environmental stressors [28].

In the same way, resistance profiles to antibiotics and/or metals in Antarctic bacteria were the focus of research during the last two decades. ARBs were reported in many different isolates, often suggesting that at least a portion of those resistances could have an origin directly related to human activity [14,29,30].

The Ross Sea, one of the most ecologically significant regions of the Southern Ocean, represents an ideal natural laboratory due to its relative isolation and limited baseline pollution. Although antibiotic and heavy metal resistance has been reported in Antarctic bacteria, most studies have focused on isolated environmental matrices, while comprehensive assessments integrating multiple matrices (seawater, sediments, and wild fish) with both phenotypic and genotypic analyses are still lacking. In particular, the extent to which human activities influence the local resistome in Terra Nova Bay remains poorly understood. Therefore, there is a clear need to investigate the co-occurrence of antibiotic and heavy metal resistance in marine bacteria across multiple environmental compartments to better understand the ecological drivers and potential anthropogenic impact on microbial communities in this relatively pristine polar ecosystem.

Nevertheless, localized contamination has been documented in proximity to the Italian research station Mario Zucchelli (MZS) in Terra Nova Bay, where sediments, seawater, and marine organisms exhibit evidence of exposure to anthropogenic stressors such as: microplastics, anthropogenic materials, heavy metals, and personal products fragrances [9,11,13,24,31].

This study examines marine bacteria isolated from seawater, sediments, and wild specimens of the Antarctic fish *Trematomus bernacchii* (Nothotenidae, Boulenger, 1902) collected in Terra Nova Bay, with a specific focus on the relationship between antibiotic resistance and heavy metal tolerance. The objectives were (i) to isolate and characterize antibiotic-resistant bacteria from multiple environmental matrices; (ii) to assess their tolerance to a broad spectrum of heavy metals; and (iii) to screen for key antibiotic resistance genes (ARGs) to identify potential mechanisms of resistance and evaluate whether human activities may be influencing the local resistome. By integrating phenotypic and genotypic analyses, this work provides new insights into the occurrence and distribution of antibiotic- and heavy-metal-resistant bacteria in a polar marine ecosystem, enhancing our understanding of the ecological implications and potential environmental origins of resistance traits in the Ross Sea.

## 2. Materials and Methods

### 2.1. Study Area

**Figure 1:**
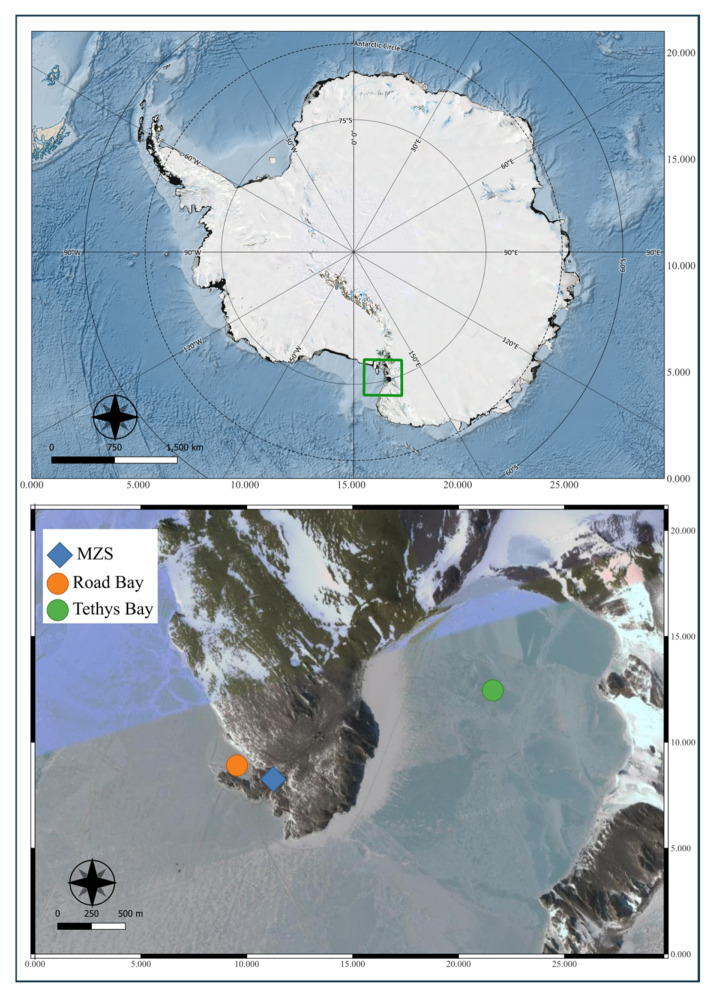
Study area (Terra nova Bay, Ross Sea). The map was generated with Quantarctica 3.2 in QGIS Münster (v. 3.16). CRS used is EPSG:3031-WGS84 Antarctic Polar Stereographic.

The Italian base, Mario Zucchelli Station (MZS), is located on a granitic headland along the coast of the Northern Foothills, northeast of Gerlache Inlet (74°41′ S, 164°06′ E)—a 7 km-wide inlet in the northwest corner of Terra Nova Bay (Ross Sea). This coastal marine area spans 29.4 km^2^ between Cape Washington and the Drygalski Ice Tongue (Figure 1). It holds significant value for long-term scientific research, and in 2003, Italy proposed its designation as Antarctic Specially Protected Area (ASPA) No. 161.

### 2.2. Samplings

Samples were collected during the 37th Italian Antarctic expedition in the austral summer of 2021–2022. Two sites were selected for sampling Site A, Road Bay (Lat. 74°41′45.9″ S, Long. 164°07′37.5″ E), located near the base, and Site B, Tethys Bay (Lat. 74°42′05.5″ S, Long. 164°02′28.6″ E). Twelve specimens of *T. bernacchii* were collected using fishing rods and artificial bait. The fish were stored in separate bags until they reached the laboratory. Seawater was collected using a Niskin bottle at three different depths: surface, 10 m and the bottom (16 m). Sediment sampling was performed using a Van Veen grab. The main chemical-physical parameters were recorded using a multi-parametric probe (Pro Plus, Hanna, Woonsocket, RI, USA).were oxygen, temperature, salinity, pH, and depth (Table S1). All samples were handled using sterile techniques to prevent cross-contamination.

### 2.3. Heterotrophic Bacterial Isolation

Initial bacterial isolations took place at the MZS laboratory. Anal swabs were taken to isolate intestinal bacteria. The collected material was inoculated onto Marine Agar (MA, Condalab, Madrid, Spain), Tryptic Soy Agar (TSA, Condalab), and Thiosulfate-Citrate-Bile salts—Sucrose (TCBS, Condalab) agar, and then incubated at 4 °C. A 0.1 mL volume from each water sample was spread onto TCBS and MA. Sediment samples (0.1 mL) were inoculated onto MA and TCBS both directly and after dilution up to 10^−3^. All bacterial strains were stored in refrigerators aboard the Italian icebreaker “Laura Bassi” during transport to Italy, where they underwent further analysis. Upon arrival, each strain was refreshed and re-isolated to obtain pure cultures. Fifty bacterial strains were selected, including twenty two from *T. bernacchii* (44%), twelve from surface water (24%), eleven from 10 m depth within the water column (22%), two from bottom water (4%), three from marine sediments (6%), and tested (see Table S2).

### 2.4. Antibiotic Susceptibility Tests

The susceptibility of Antarctic heterotrophic marine bacteria to antibiotics was assessed using the agar disc diffusion method described previously by Bauer et al. [32] and according to [33]. The selected bacterial strains were tested against 28 antibiotic discs (Table S2). These antibiotics were chosen to represent various classes based on their mechanisms of action. The antimicrobial agents tested included: Cellular wall inhibitors: (1) Cephalosporins 1st generation (Cefalexin, CL, 30 μg), Cephalosporins 2nd generation (Cefoxitin, FOX, 30 μg), and Cephalosporins 3rd generation (Cefotaxime CTX, 30 μg); (2) Peptidoglycan inhibitors: Beta-lactams: Fosfomycin (FOS, 50 μg), Oxacillin (OX, 5 μg), Penicillin G (P, 10 μg), Amoxicillin (AML, 10 μg), Piperacillin (PRL, 30 μg); (3) Glycopeptide antibiotics: Vancomycin (VA, 30 μg), Polymyxin B (PB 300 μg) and amoxicillin + Clavulanic acid (AMC, 3 μg); (4) Nucleic acid inhibitors: (1) Quinolones: (Levofloxacin, LEV, 5 μg), (2) Fluoroquinolones (Ciprofloxacin, CIP, 5 μg), (Flumequine, UB, 5 μg); (5) Potentiated sulphonamides (Sulphathiazole + Trimethoprim, SSXT, 25 μg). RNA synthesis inhibitors: Rifampicin (RD, 30 μg, RA 5 μg,), Novobiocin (NV, 5 μg), Nitrofurans (Nitrofurantoin F, 200 μg); (6) Protein synthesis inhibitors: (a) Lincomycin (Clindamycin, CD, 10 μg); (b) Aminoglycosides (Gentamicin, CN, 30 μg), Kanamycin (K, 30 μg), Tobramycin (TOB, 10 μg); (c) Macrolides (Erythromycin, E, 15 μg) Azithromycin (AZM, 15 μg), Neomycin (N, 30 μg); (d) Tetracyclinides (Tetracycline, TE, 30 μg); (e) Amphenicols (Chloramphenicol, C, 30 μg). The antibiotic discs were purchased from Condalab. Agar plates were inoculated with a suspension of each bacterial strain at a concentration of 0.5 McFarland standard. The discs were then placed on the agar Petri dishes for 96 h at 4 °C. Following the guidelines of the [33], at least two control plates were included for each bacterial strain tested for antimicrobial susceptibility: one without antimicrobial agents (positive control) and one without bacterial strains (negative control). The absence of bacterial growth around the antibiotic disc indicated that the bacterium was sensitive to the antibiotic. Results were reported according to Tomova et al. [14] with the following sensitivity grades: S = sensitive (≥21 mm), I = intermediate (16–20 mm), and R = resistant (≤15 mm). Moreover, bacteria showing multiple antibiotic resistance were tested for heavy metal resistance.

### 2.5. Multiple Antibiotic Resistance (MAR) Index

To investigate the distribution of antibiotic resistance in the study area, the MAR index was calculated.

This is given by the formula

MAR index=a/b

where “a” is the number of antibiotics tested to which each bacterial strain was resistant and “b” is the total number of antibiotics against which each single bacterial strain was tested [34]. The MAR index is categorized as high (≥0.6), moderate (0.21–0.59), and low (≤0.2).

### 2.6. Heavy Metals Test in Antibiotic-Resistant Bacterial Strains

The metal tolerance of the isolates was evaluated using the plate diffusion method, according to the protocol described by Arcadi et al. [35] with slight modifications. Eight heavy metal salts were tested in this study: Lead(II) acetate trihydrate (Pb(C_2_H_3_O_2_)_2_·3H_2_O), Cadmium sulphate hydrate (3CdSO_4_·8H_2_O), Nickel chloride (NiCl_2_), Cobalt(II) chloride hexahydrate (Cl_2_Co·6H_2_O), Copper(II) sulphate pentahydrate (CuSO_4_·5H_2_O), Mercury(II) chloride (HgCl_2_), Zinc sulphate heptahydrate (ZnSO_4_·7H_2_O), and Potassium chromate (K_2_CrO_4_). All metal salts were sourced from Sigma-Aldrich (St. Louis, MO, USA). In brief, aliquots (10 mL) of each metal salt dissolved in filter-sterilized phosphate-buffered saline (PBS, 0.5 mL, pH 6.8) added to a central well in each agar plate. The bacterial suspensions were inoculated in radial streaks in each plate and incubated at 4 °C for 96 h. Each test was performed in two replicates, in addition PBS with no metal that served as the negative control. The tested concentrations were 10, 100, 1000, 5000, and 10,000 ppm. After incubation time, metal tolerance of each bacterial strain was evaluated by measuring the radial growth and comparing it to the respective negative control. Bacterial resistance was assessed by calculating the percentage of the growth length (mm) to the total length of the inoculated streak. The results were reported as percentage values: 100% very resistant (VR, 50–45.6 mm), 75% resistant (R, 45.5–25.6 mm), 25% slightly resistant (SR, 25–6 mm), and 0% sensitive (S, 5–0 mm).

### 2.7. Multiple Heavy Metal Resistance (MHMR) Index

The MHMR index, following a method similar to the MAR index, was determined by calculating the ratio of resistant isolates to the total number of heavy metals tested. An MHMR value greater than 0.2 indicates a high risk of heavy metal contamination, whereas an MHMR index below 0.2 suggests a lower risk of environmental pollution [36].

### 2.8. Screening of ARGs and Taxonomic Identification

A total of 48 marine bacterial isolates were screened by PCR for the presence of genes encoding antibiotic resistance (TS3). Genes selected for analysis in this research include *TET* (tetracycline), *VAN* (vancomycin), *SULF* (sulphonamide), *qacB* (quaternary ammonium compound), *qnrA* (quinolone resistance), and *oqxB* (quinolone efflux pump). The list of primers used in this study (Eurofins, genomic Italy srl, Vimodrone, Italy), with details sequence and annealing temperature, is reported in Table S4. Twelve bacterial strains were selected and identified taxonomically through Sanger 16S rRNA gene sequence analysis.

The bacteria gDNA was extracted using the PCRBIO Rapid Extract Lysis Kit (PCR Biosystems Inc., Wayne, PA, USA). The gDNA was used as the template to amplify the 16S rRNA genes with primers p8FPL (5′-AGTTTGATCCTGGCTCAG-3′) and 1541R (5′-AAGGAGGTGATCCAGCCGCA-3′) [37] using Platinum™ PCR SuperMix High Fidelity (Thermo Fischer Scientific, Waltham, MA, USA). The PCR was carried out under the following thermocycling conditions: 95 °C for 2 min, followed by 30 cycles of 95 °C for 60 s, 55 °C for 60 s, 68 °C for 90 s, and a final extension at 68 °C for 7 min. Obtained amplicons were tested on agarose gel (1.5%), purified with ExoSAP (Thermo Fischer Scientific) and full-length sequenced in a ABI 3730XL DNA Analyzer (the assay was performed by BMR Genomics, Padua, Italy).

Raw sequences were checked for possible chimeric origin using the Pintail 1.1 software [38]. Alignment of the amplified 16S rRNA gene sequences and of the closest relative identified with BLAST [39] was initially made with Clustal Omega algorithm [40] (Sievers and Higgins, 2021), followed by manual alignment within Sea View Version 4 [41].

The phylogenetic tree based on distance analysis, was generated using the neighbor-joining algorithm and the Jukes–Cantor distance matrix, and one thousand bootstrap re-samplings were performed to estimate the robustness of the tree using the same distance model always within Sea View Version 4. The sequences were deposited into the GenBank database under the accession numbers PV018936–PV018947.

## 3. Results

### 3.1. Antibiotic-Resistant Bacteria

**Figure 2:**
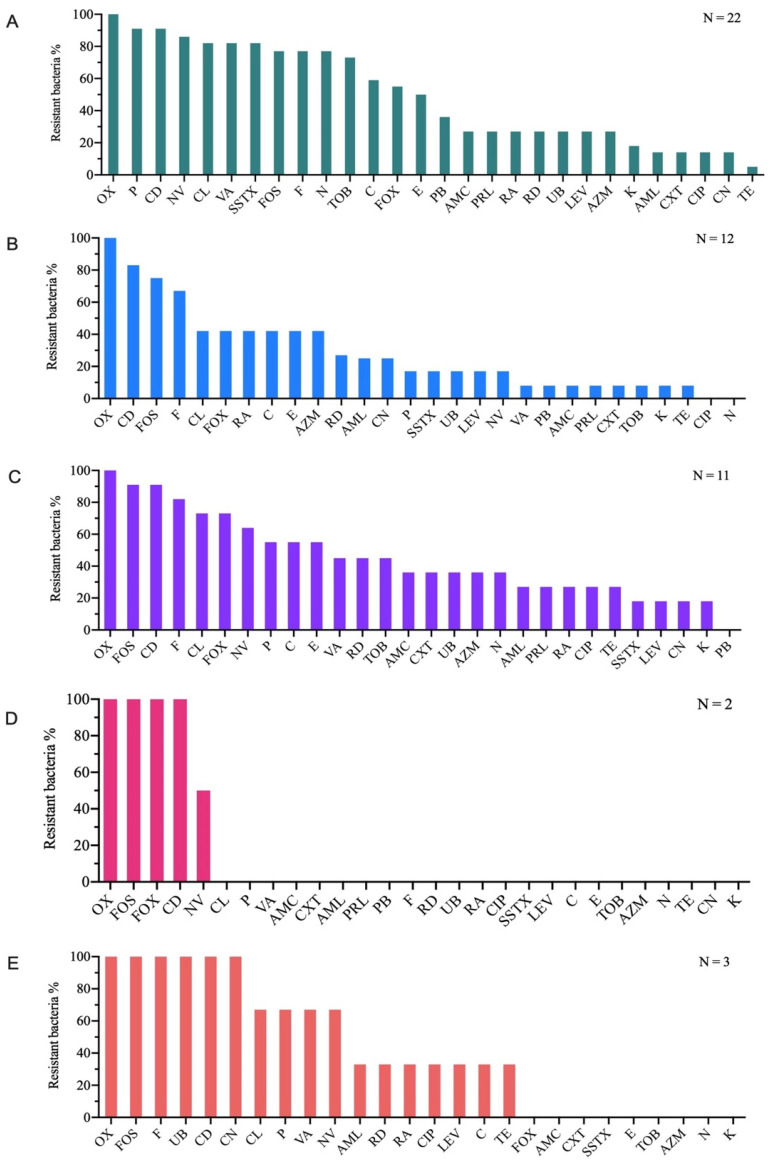
Percentages of bacterial strains, isolated from *Trematomus bernacchii* (**A**). Percentages of bacteria, isolated from superficial water (**B**). Percentages of bacteria, isolated from 10 m depth along the water column (**C**). Percentages of bacteria, isolated from bottom water (**D**), and percentages of bacterial strains isolated from sediments (**E**) resistant to tested antibiotics.

All the heterotrophic marine bacteria isolated from *T. bernacchii* specimens exhibited multidrug resistance, ranging from 6 to 19 antibiotics. The resistance percentages ranged from 21% to 68%. Specifically, 100% of the strains were resistant to OX, 91% to P and CD, 86% to NV, and 82% to VA, SSXT, and CL. Additionally, 77% showed resistance to N, FOS, and F, while 73% were resistant to TOB, 59% to C, 55% to FOX, and 50% to E (see Figure 2A).

The bacteria isolated from surface water exhibited multidrug resistance, ranging from 14% to 64%, corresponding to resistance against 4 to 18 antibiotics. The antibiotic susceptibility test indicated that all marine bacterial isolates were resistant to OX (100%), with 83% also resistant to CD, 75% to FOS, and 67% to F. In contrast, the isolates were sensitive to CIP and N (Figure 2B). Likewise, strains collected from a 10-m depth in the water column exhibited multidrug resistance, with resistance levels ranging from 29% to 61%, corresponding to resistance against 8 to 17 different antibiotics.

All bacterial isolates exhibited resistance to OX, with 91% also resistant to CD and FOS, 82% to F, 73% to CL and FOX, and 64% to NV. Furthermore, 55% showed resistance to C, E, and P, while none of the strains demonstrated resistance to PB (Figure 2C).

Regarding bacteria from bottom water, they demonstrated multidrug resistance ranging from 14% to 18%, corresponding to resistance against 4 to 5 antibiotics. All strains were 100% resistant to OX, CD, FOS, and FOX, while 50% resisted to NV. However, they remained sensitive to all other antibiotics (Figure 2D). Finally, strains isolated from sediments showed multidrug resistance ranging from 32% to 54%, corresponding to resistance against 9 to 15 antibiotics. All strains (100%) were resistant to CD, FOS, F, UB, CN, and OX; 67% resisted to CL, NV, P, and VA; and 33% to C, RD, RA, AML, TE, CIP, and LEV. The bacteria were sensitive to the remaining antibiotics (Figure 2E).

**Figure 3:**
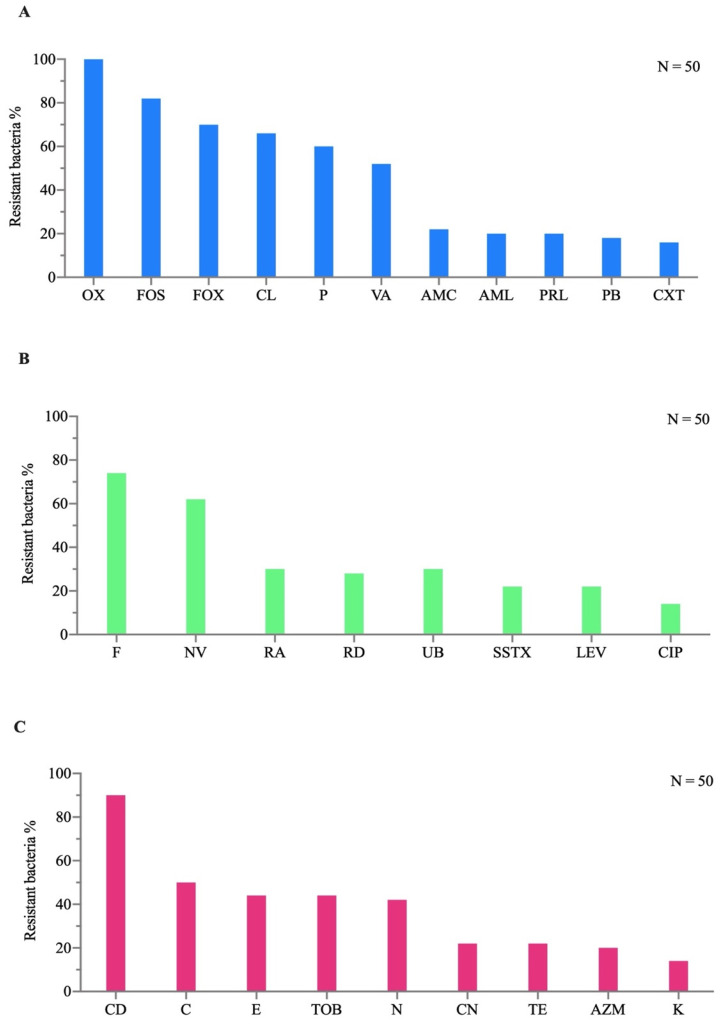
Percentages of bacteria tolerant to cell wall inhibitors antibiotics (**A**), to nucleic acid inhibitors antibiotics (**B**), and to protein synthesis inhibitors antibiotics (**C**).

The results showed that all tested strains exhibited multi-resistance to antibiotics. Specifically, 62% (31 strains) were resistant to 10 to 19 antibiotics, while the remaining 38% (19 strains) showed multi-resistance to 4 to 9 antibiotics. Additionally, 18 strains demonstrated a multi-resistance rate between 50% and 63%. The strains were evaluated for resistance to cell wall-inhibiting antibiotics, showing complete resistance to OX (100%), across all strains. Furthermore, 82% of strains resisted to FOS, 70% to FOX, 66% to CL, 62% to NV, 60% to P, and 52% to VA (Figure 3A). Bacterial strains tested against nucleic acid inhibitor antibiotics showed the highest resistance rates ranging from 14% for CIP to 74% for F (Figure 3B). Finally, for protein synthesis inhibitors, 90% of the strains exhibited tolerance to CD and 50% were tolerant to C (Figure 3C).

The MAR index values indicated the proportion of antibiotic-resistant (AR) bacteria in the study area, ranging from 0.14 to 0.68. Most bacteria (74%) showed a moderate MAR index between 0.29 and 0.57, while 14% had a high MAR index, and 12% displayed a low MAR index.

### 3.2. Heavy Metals

**Figure 4:**
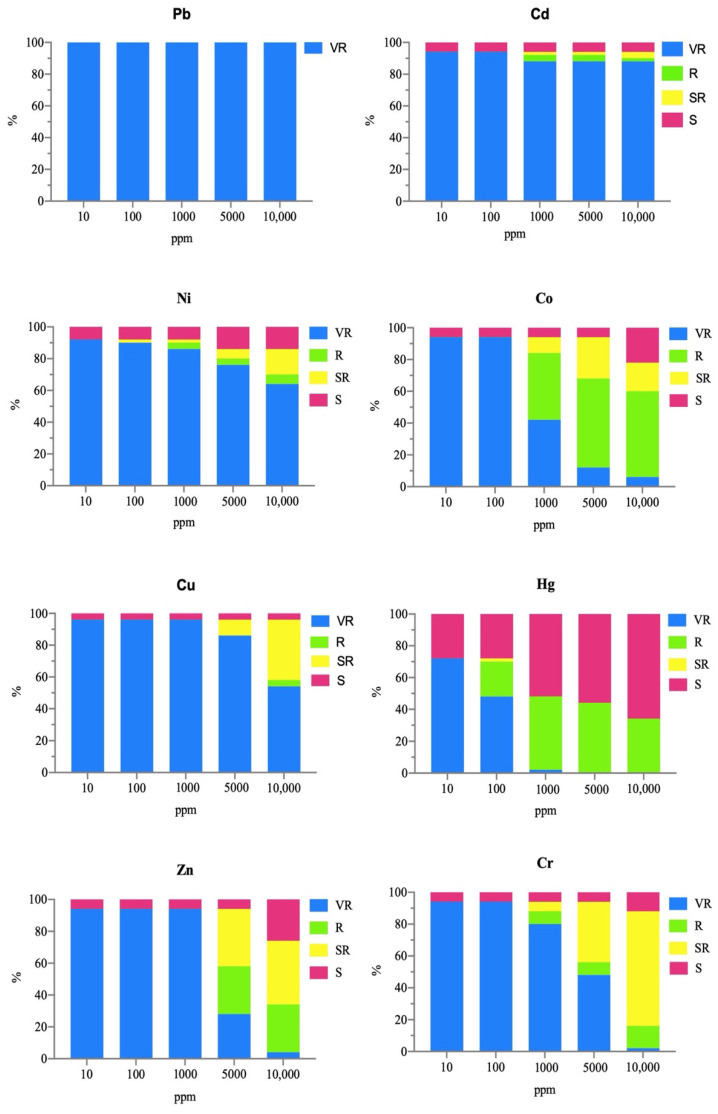
Percentage of bacterial resistance to Pb, Cd, Ni, Co, Cu, Hg, Zn, and Cr at concentrations of 10, 100, 1000, 5000, 10,000 ppm. VR: very resistant, R: resistant, SR: slightly resistant, S: sensitive.

The results at a concentration of 10 ppm, showing resistance levels in the bacteria ranging from 74% to 98%, are presented in Figure 4. At a concentration of 10,000 ppm, 98% of the strains exhibited the following resistance trend: Pb > Cd > Ni > Co > Cu > Hg > Zn > Cr.

Specifically, the resistance percentages to the various metal salts were as follows: 90% for Pb, 68% for Cd, 60% for Ni, 54% for Co, 38% for Cu, 38% for Hg, 34% for Zn, and 20% for Cr (Figure 4). The results showed that all isolates (100%) were resistant to lead at every concentration tested. For cadmium, 94% of the bacteria were highly resistant at 10 ppm, while at 10,000 ppm, 88% remained highly resistant (Figure 4). Regarding nickel, 92% of the isolates tested at 10 ppm were highly resistant, whereas only 64% remained highly resistant at 10,000 ppm. Regarding cobalt, the percentage of highly resistant bacteria was 94% at 10 ppm, but it gradually decreased with increasing metal concentration, dropping to 6% at 10,000 ppm. Bacterial strains exhibited high resistance (96%) to copper at 10 ppm, but at the higher concentration, resistance dropped to 54%. For mercury, 72% of bacteria were highly resistant at 10 ppm, but starting from 5000 ppm, they became more sensitive. For zinc and chromium, the percentage of highly resistant bacteria, which was 94% at 10 ppm, gradually decreased with increasing metal concentration, reaching only 4% and 2% at 10,000 ppm, respectively.

The MHMR index results indicated that at the maximum heavy metal concentration of 10,000 ppm, the metal resistance index ranged from 0.1 to 1. Among the bacterial strains, 30% exhibited an index of 0.8, 26% had an index of 1, 22% recorded 0.6, and 16% showed 0.8. Only 2% had an index below 0.2. At a concentration of 10 ppm, the MHMR index showed that all bacteria had values above 0.2. Selected bacteria displayed very high MHMR index values, all ranging between 0.6 and 1.

### 3.3. Bacterial Taxonomy

Twelve bacterial strains were selected and taxonomically identified by Sanger 16S rRNA gene. The strains were selected for sequencing according to predefined criteria, including high multidrug resistance, tolerance to multiple heavy metals, and the presence of antibiotic resistance genes detected by PCR, in order to identify those most relevant to the objectives of the study.

**Figure 5:**
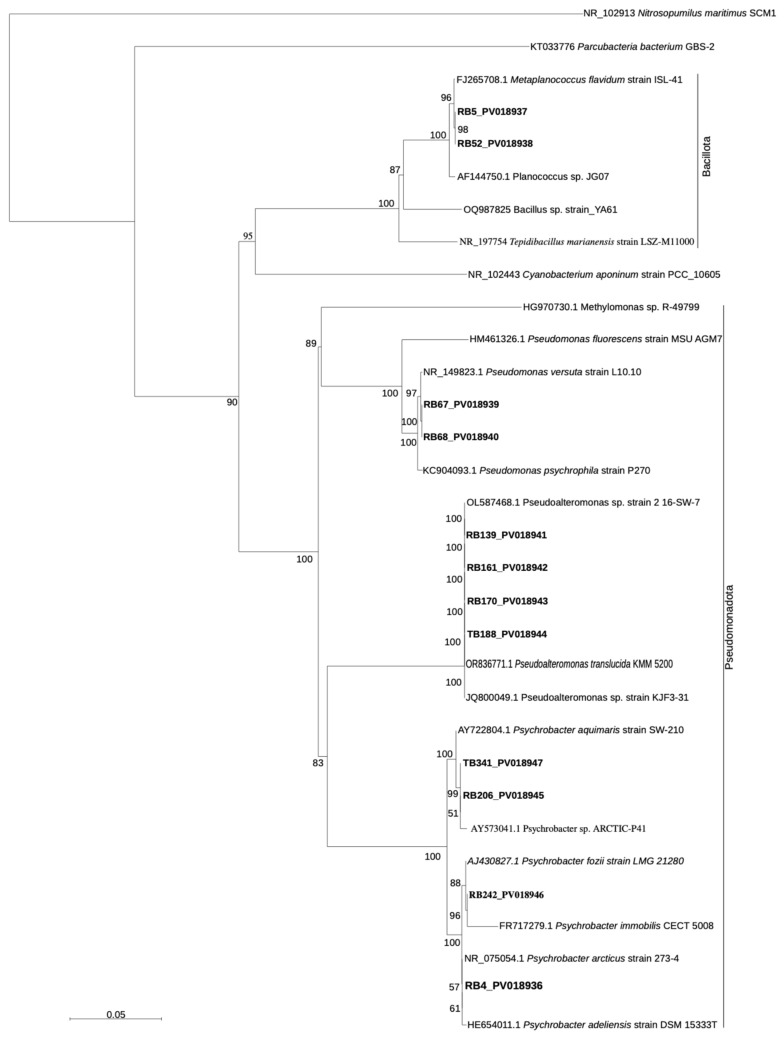
Rooted phylogenetic tree clustered by neighbour-joining of maximum likelihood values showing the affiliation from the bacterial 16S rRNA gene sequences sourced from the strains to closest-related sequences from members of different bacterial phylum. Isolates obtained within this research are indicated in bold type. Percentages of 1000 bootstrap resampling that supported the branching orders in each analysis are shown above or near the relevant nodes (only values P50% are shown), sequence of *Nitrosopumilus maritimus* SCM1 was employed as an out-group.

On the basis of taxonomic affiliation, 2 strains, RB5 and RB52, were assigned to the *Bacillota phylum*, with 98.88% similarity to the strain *Metaplanococcus flavidum*_ISL-41 (FJ265708). The remaining 10 isolates belonged to the phylum Pseudomonadota, two isolates namely RB67 and RB68 belonged to the family Pseudomonadaceae and showed 100% similarity with strain L10 of *Pseudomonas versuta*, four isolates (RB139, RB161, RB170, and TB188) belonged to the family Pseudoalteromonadaceae with more than 99% similarity to *Pseudoalteromonas translucida* KMM 520, and, finally, the last four strains TB341, RB206, RB242, and RB4 belonged to the family *Moraxellaceae*, to three different genera of *Psychrobacter* with similarity exceeding 99% (see Figure 5).

### 3.4. Screening of AR Genes

Table 1 provides an overview of the isolates obtained from *T. bernacchii*, surface seawater, the water column and marine sediments, together with their corresponding antibiotic resistance gene (ARG) profiles. From *T. bernacchii*, seven bacterial isolates were identified. Two strains were assigned to *Pseudomonas versuta* (RB67 and RB68) and exhibited an identical ARG complement, comprising *VAN*, *SULF*, *qacB*, *qnrA* and *oqxB*. Three isolates belonged to *Pseudoalteromonas translucida* (RB139, RB161, and RB170), all carrying *VAN*, *SULF*, *qacB* and *oqxB*; qnrA was additionally detected in strains RB161 and RB170. Two isolates were classified as *Psychrobacter* spp. (RB206 and TB341), both harbouring TET, *VAN*, *SULF*, *qacB* and *oqxB*. Surface seawater yielded two strains: *Psychrobacter* sp. RB4, positive for *TET*, *VAN*, *SULF*, *qacB* and *oqxB*, and *Metaplanococcus flavidum* RB5, which harboured *SULF* and *oqxB*. Two further isolates were recovered from the water column. *M. flavidum* RB52 carried *VAN*, *qacB* and *oqxB*, whereas *Psychrobacter* sp. RB242 displayed the same ARG set (*TET*, *VAN*, *SULF*, *qacB*, and *oqxB*) observed in the surface water isolate RB4. Finally, one sediment-derived isolate (TB188), identified as *P. translucida*, possessed *TET*, *qacB*, *qnrA* and *oqxB* (Table 1).

### 3.5. Antibiotic and Heavy Metals Resistance Related to the Identified Bacterial Strains

Among the strains isolated from *T. bernacchii*, the strain RB67 (*P. versuta*) exhibited resistance to 13 antibiotics, with the majority targeting cell wall inhibitors (6 antibiotics), resulting in a MAR index of 0.46. Similarly, RB68 (*P. versuta*) was resistant to 11 antibiotics, with 6 of them targeting cell wall inhibitors, yielding a MAR index of 0.39. Both strains exhibited resistance to all tested metals at all concentrations, except for Hg, to which it was resistant up to a concentration of 100 ppm.

The strain RB139 (*P. translucida*) exhibited resistance to 11 antibiotics, with the majority (6 out of 11) targeting cell wall inhibitors, resulting in a MAR index of 0.39. It was resistant to all tested heavy metals and concentrations, except for HgCl_2_, where resistance was observed up to 1000 ppm.

The strain RB161 (*P. translucida*) displayed resistance to 12 antibiotics, half of which were cell wall inhibitors, with a MAR index of 0.43. It was resistant to all tested heavy metals and concentrations, except for HgCl_2_, where resistance was observed up to 1000 ppm.

**Figure 6:**
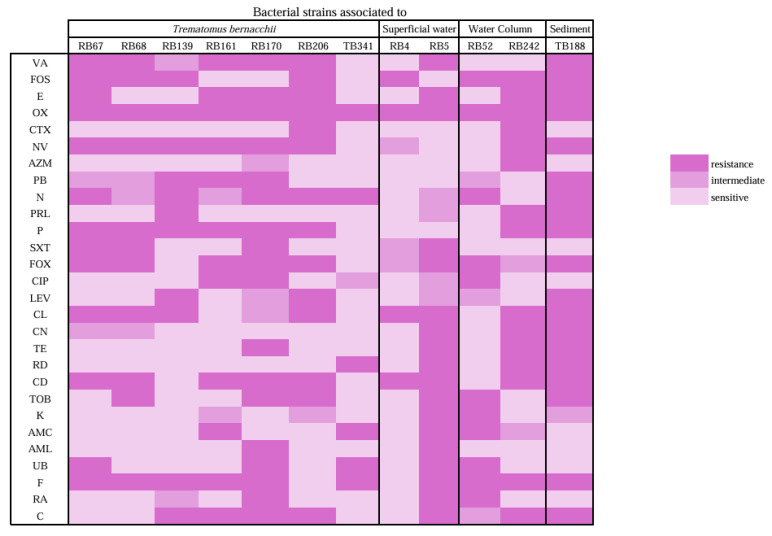
Twelve selected bacterial strains associated with *Trematomus bernacchii*, superficial water, column water and sediment, selected against 28 antibiotics; R: resistant, I: intermediate, S: sensitive.

The RB170 (*P. translucida*) demonstrated resistance to 18 antibiotics, evenly distributed among cell wall inhibitors (6), nucleic acid inhibitors (6), and protein synthesis inhibitors (6), with a MAR index of 0.64. It exhibited resistance to all tested heavy metals at all concentrations, except for Hg, where resistance was observed up to 100 ppm (Figure 6, Table S3). The strain RB 206 (*Psychrobacter* sp.) exhibited resistance to 14 antibiotics, with 7 out of 14 targeting cell wall inhibitors, resulting in a MAR index of 0.5. This strain demonstrated multi-resistance to Pb, Cd, Co, Cu, and Cr at all tested concentrations, while resistance to Hg was observed up to 100 ppm (Figure 6, Tables S3 and S5). And finally, the strain TB341 (*Psychrobacter* sp.) was resistant to six antibiotics, with 4 out of 6 being cell wall inhibitors, and had a MAR index of 0.21. It exhibited multi-resistance to Pb, Cd, Co, Cu, and Cr at all tested concentrations, while resistance to Hg was observed up to 1000 ppm (Figure 6, Table S3). The two bacterial strains isolated from surface water samples were *Psychrobacter* sp. (RB4) and *Metaplanococcus flavidum* (RB5). *Psychrobacter* sp. (RB4) RB4 exhibited resistance to four antibiotics, three of which were cell wall inhibitors, with a MAR index of 0.14. This strain displayed multi-resistance to Pb, Cd, Co, Cu, and Cr at all tested concentrations. It was resistant to Ni salt up to 1000 ppm and to Zn salt up to 5000 ppm, while it remained sensitive to Hg at all concentrations. *M. flavidum* (RB5) was resistant to 18 antibiotics, with the majority targeting protein synthesis inhibitors (7 out of 18) and cell wall inhibitors (6 out of 18), resulting in a MAR index of 0.64. This strain exhibited resistance to Pb, Cd, Cu, Zn, and Cr at all tested concentrations, while resistance to Co salt was observed up to 5000 ppm and to Ni and Hg up to 1000 ppm.

Two bacterial strains isolated from column water, *M. flavidum* (RB52) and *Psychrobacter* sp. (RB242) exhibited varying levels of antibiotic and heavy metal resistance. *M. flavidum* (RB52) exhibited resistance to 11 antibiotics, including 4 targeting cell wall inhibitors, 4 nucleic acid inhibitors, and 3 protein synthesis inhibitors, with a MAR index of 0.39. Additionally, it was resistant to all tested metals at all concentrations, except for Hg, to which it was sensitive at concentrations up to 100 ppm. *Psychrobacter* sp. (RB242) was resistant to 15 antibiotics, including 6 targeting cell wall inhibitors and 6 protein synthesis inhibitors, with a MAR index of 0.53. It displayed multi-resistance to Pb, Cd, Co, Cu, and Cr at all tested concentrations, while resistance to Hg salt was observed only up to 1000 ppm (Figure 6). Finally, *P. translucida* (TB188), a strain isolated from marine sediments, exhibited resistance to 9 antibiotics, including 4 targeting cell wall inhibitors and 3 nucleic acid inhibitors, with a MAR index of 0.32. It was resistant to all tested heavy metals and concentrations, except for Hg salt, where resistance was observed up to 5000 ppm (Figure 6, Table S3).

## 4. Discussion

### 4.1. Antibiotic-Resistant Bacteria

The detection of antibiotic resistance in marine bacterial strains isolated from fish, seawater, and sediment in Terranova Bay may be linked to the discharge of treated wastewater into the area.

Previous studies indicate that biological wastewater treatment plants create conditions that promote the development and spread of antibiotic resistance by combining key factors such as high microbial density in nutrient-rich environments and recurrent exposure to antibiotics and resistant bacteria [42,43].

Our results demonstrate that wild fish from the Ross Sea host antibiotic-resistant bacteria (ARBs) carrying genes that confer resistance to antibiotics commonly used in both human and veterinary medicine.

As data on antibiotic resistance in Antarctic fish remain scarce, we compared our findings with studies conducted on other Antarctic marine organisms. Notably, Mangano et al. [44] reported that 64.2% of bacterial strains isolated from sponges in Terra Nova Bay were resistant to tetracycline (TE), whereas resistance to TE in our fish-derived isolates was markedly lower (5%). Conversely, resistance to kanamycin (K) and chloramphenicol (C) in sponge-associated bacteria ranged from only 1.5% to 7.5% [45], while our isolates showed substantially higher resistance levels, ranging from 18% to 59%. Similarly, resistance to tobramycin (TOB), polymyxin B (PB), penicillin (P) and novobiocin (N) in sponge-associated bacteria varied between 23.9% and 40.3% [44], whereas fish-associated strains in our study exhibited consistently higher rates, reaching 36% for PB and up to 91% for P.

Further differences emerged when comparing our data with those reported by González-Aravena et al. [45], who found that bacteria isolated from Sterechinus neumayeri, from Fildes Peninsula, were fully susceptible to SSTX and showed lower resistance to chloramphenicol (34%), cefotaxime (CTX; 21%), kanamycin (8%), gentamicin (CN; 8%) and tetracycline (3%) than observed in our fish isolates (82%, 59%, 14%, 14% and 5%, respectively).

Comparable resistance patterns have also been documented in Antarctic wildlife. Retamal et al. [46] and Na et al. [47], in Fildes Peninsula, detected bacterial strains resistant to amoxicillin (AML), cefotaxime (CTX), gentamicin (CN), ciprofloxacin (CIP), chloramphenicol (C) and tetracycline (TE) in penguin faeces from the Antarctic and Fildes Peninsulas. Gutiérrez et al. [3] reported variable resistance to gentamicin (CN) and erythromycin (E) in birds and mammals, with *Leptonychotes weddellii* and *Arctocephalus gazella* (Fildes Penisula) showing the highest resistance levels (100% and 88%, respectively), whereas fish isolates in our study exhibited moderate resistance (CN 14%, E 50%).

Overall, resistance to β-lactams appears to be more widespread than resistance to aminoglycosides across Antarctic environments [25]. Bacteria isolated from King George Island have shown resistance to multiple antibiotic classes, including β-lactams, macrolides, aminoglycosides, sulphonamides, cephalosporins, polymyxins and fluoroquinolones [30,43,47,48,49,50]. In line with these findings, Hernández et al. [30] reported particularly high resistance to ampicillin (AMP), sulfamethoxazole/trimethoprim (SXT), cefoxitin (FOX), streptomycin (S), nalidixic acid (NAL) and cefotaxime (CTX) in bacteria from Fildes Bay, further highlighting the significance of our results within the broader Antarctic context.

Our results highlight a distinct and previously undocumented pattern of antibiotic resistance in bacteria associated with Antarctic fish from the Ross Sea. Compared with earlier studies on Antarctic sponges, echinoderms and wildlife, fish-associated bacteria showed higher resistance to several antibiotics, particularly β-lactams and chloramphenicol, while maintaining low resistance to tetracycline. Importantly, our findings demonstrate that antibiotic resistance is not restricted to higher trophic levels or coastal wildlife but is also well established in fish, highlighting their role as an under-recognised reservoir within Antarctic marine ecosystems.

With regard to water samples, De Souza et al. [29] reported higher resistance to chloramphenicol (C; 63%) than that observed in our study, whereas resistance to tetracycline (TE; 11%) and kanamycin (K; 6%) was comparable to our findings. In contrast, our water-derived isolates showed lower resistance to C, indicating site-specific differences in resistance patterns. Regarding sediments, Lo Giudice et al. [51], in bacterial strains from Terra Nova Bay sediments, detected resistance to both chloramphenicol (C) and kanamycin (K) whereas sediment isolates in our study remained fully susceptible to kanamycin, highlighting a key difference between the two areas.

Further contrasts emerged when comparing our results with those of Tomova et al. [14], who investigated antibiotic-resistant bacteria on Deception Island and Galindez Island and reported high resistance levels to erythromycin (E; 62%), chloramphenicol (C; 58%), vancomycin (VA; 58%) and tetracycline (TE; 42%) in maritime Antarctic samples. In comparison, our sediment-derived isolates were fully susceptible to erythromycin but showed resistance to chloramphenicol and tetracycline (both 33%) and particularly high resistance to vancomycin (67%), indicating a distinct resistance profile in Ross Sea sediments.

Similarly, Na et al. [47] reported substantially higher resistance to ciprofloxacin (CIP; 48–54%) in bacteria from the Fildes Peninsula than that observed in our study (33%), further emphasising the lower fluoroquinolone resistance detected in our samples.

Overall, these differences are likely to reflect variations in local environmental conditions, antibiotic exposure and human impact. Although antibiotic resistance genes (ARGs) may occur naturally in Antarctic environments [52], growing evidence suggests that human activities can significantly increase their prevalence [53]. In this context, our results, obtained from a comparatively less impacted area, underscore the heterogeneity of the Antarctic resistome and support the hypothesis that proximity to human activities influences resistance patterns. Jara et al. [54] demonstrated that bacterial communities near research stations in the Fildes Peninsula exhibit greater tolerance to synthetic antibiotics, likely due to wastewater discharges that introduce mobile genetic elements such as transposons, integrons and plasmids, a mechanism that appears less pronounced in the Ross Sea according to our findings.

### 4.2. Heavy Metal-Resistant Bacteria

Inorganic contaminants in Antarctica are primarily composed of heavy metals, including copper (Cu), lead (Pb), mercury (Hg), chromium (Cr), and cadmium (Cd) [55]. Human activities have resulted in localized metal accumulation, particularly around scientific stations, involving lead (Pb), copper (Cu), arsenic (As), nickel (Ni), and zinc (Zn) [7,56,57].

As no published data are available on metal tolerance in bacteria associated with Antarctic fish, it was not possible to compare our results with those from other fish species. Consequently, our findings were compared with studies conducted on other marine organisms and environmental matrices from the same or comparable Antarctic areas. Mangano et al. [44] reported that bacterial isolates from Antarctic sponges collected near MZS exhibited complete tolerance to cadmium (Cd) up to 1000 ppm and resistance patterns to mercury (Hg) similar to those observed in our study, although only 22% and 16% of sponge-associated bacteria tolerated Hg at 2500 and 5000 ppm, respectively. While all sponge isolates tolerated zinc (Zn) at 2500 ppm, tolerance decreased markedly at higher concentrations. In contrast, all bacterial isolates obtained in our study showed full tolerance to Cd at all tested concentrations, and a substantially higher proportion (68%) tolerated Hg up to 5000 ppm. Consistent with Mangano et al. [44], strains identified as *Pseudoalteromonas translucida* in our study displayed resistance to metal concentrations as high as 10,000 ppm.

Mangano et al. [44] reported that bacteria from Antarctic sponges near MZS exhibited complete tolerance to Cd up to 1000 ppm, similar to their resistance to Hg, although only 22% and 16% tolerated Hg at 2500 and 5000 ppm, respectively. All isolates resisted Zn at 2500 ppm, but tolerance decreased with increasing concentrations (30% at 5000 ppm, 21% at 7500 ppm, and 18% at 10,000 ppm).

In contrast, our isolates showed full resistance to Cd at all tested concentrations, while 68% tolerated Hg up to 5000 ppm. As previously observed by Mangano et al. [44], *Pseudoalteromonas translucida* strains exhibited resistance up to 10,000 ppm. Similarly, González-Aravena et al. [45] reported tolerance to Hg and Zn in Flavobacterium spp. isolated from sea urchins in the Fildes Peninsula supporting the widespread occurrence of metal tolerance among Antarctic marine bacteria.

For what concerns water isolates, De Souza et al. [29], showed tolerance to Hg (68% at 10 ppm), Cd and Zn (29% at 100 ppm) and Cr (16% at 100 ppm), while our isolates exhibited markedly higher resistance levels. Specifically, 23% of our strains tolerated Hg at concentrations up to 10,000 ppm, all isolates tolerated Cd, 50% tolerated Zn and as many as 86% were resistant to chromium (Cr) at the same concentration. Bacteria isolated from Ross Sea sediments have previously been shown to tolerate Cd, Cu, Zn and Hg, reflecting local metal availability [51]. Our results strongly support and extend these observations, as sediment-derived isolates exhibited complete tolerance to Cd, Cu and Zn, and 33% resistance to Hg up to 5000 ppm. Comparable resistance patterns to Pb, Cu, Ni and Cr were also reported by Tomova et al. [14] in marine sediments near the Ukrainian Research Station. Finally, Bargagli et al. [58] documented cadmium accumulation in Trematomus spp. and marine sediments, likely linked to deep-water upwelling processes. In this context, the consistently high Cd tolerance observed in fish-, water- and sediment-derived bacterial strains in our study likely reflects natural adaptation to elevated Cd levels in the coastal environment of Terra Nova Bay [59]. This interpretation is further supported by the high MAR and MHMR index values calculated at both 10,000 and 10 ppm, which collectively indicate a noteworthy level of environmental metal exposure in the study area.

The occurrence of antibiotic-resistant and metal-tolerant bacteria in Antarctic environments likely reflects the interplay between long-standing natural selective pressures and localized anthropogenic influences [60]. Environmental resistance traits may be supported by intrinsic mechanisms such as efflux systems, metal sequestration, and oxidative stress response pathways, which are widespread among environmental bacteria and can be selected under natural geochemical conditions [61]. Importantly, several of these mechanisms are known to be genetically or functionally linked to antibiotic resistance through co-regulation or co-localization on mobile genetic elements, providing a mechanistic basis for co-selection in the absence of direct antibiotic pressure [62]. Consistent with this framework, antibiotic resistance genes are increasingly recognized as part of an ancient environmental resistome rather than being exclusively associated with clinical antibiotic use. In the vicinity of Antarctic research stations, additional factors may locally modulate these resistance patterns [63,64,65]. Wastewater discharge, artificial substrates, and microplastic particles can increase microbial density, promote biofilm formation, and facilitate horizontal gene transfer, thereby enhancing the persistence and spread of resistance determinants. Heavy metals detected in Antarctic matrices may further contribute to this process by exerting selective pressure that favours bacteria carrying both metal tolerance and antibiotic resistance genes [66,67,68,69]. Nevertheless, available evidence indicates that such effects are spatially restricted to areas close to human infrastructure and do not imply widespread or continent-scale alteration of Antarctic microbial communities [68,69]. Overall, human activity in Antarctica appears to act as a localized modifier of resistance dynamics, superimposed on a broader resistome shaped predominantly by natural environmental selection.

### 4.3. Antibiotic Resistance Genes (ARGs)

Antarctic environments are increasingly recognized as reservoirs of antibiotic resistance genes (ARGs), shaped by a combination of natural selective pressures and human-derived inputs [3]. In our isolates, the ARGs most frequently detected were *TET*, *VAN*, *SULF*, *qacB*, *qnrA* and *oqxB*, indicating resistance mechanisms spanning tetracyclines, glycopeptides, sulphonamides, quinolones and efflux-mediated multidrug resistance. ARG surveys in Antarctic marine bacteria remain scarce, and, to our knowledge, no previous work has investigated ARGs in fish-associated microbiota from the Ross Sea. Our study therefore provides novel evidence that *T. bernacchii*, a key benthic fish species, can host bacterial strains carrying multiple ARGs. In particular, *Psychrobacter* spp. from fish samples harboured *TET*, *VAN*, *SULF*, *qacB* and *oqxB*, representing broad resistomes consistent with their multidrug-resistant phenotypes. *Pseudomonas versuta* isolates also carried *VAN*, *SULF*, *qacB*, *qnrA* and *oqxB*, highlighting the co-occurrence of quinolone resistance and efflux-associated determinants. Similarly, *Pseudoalteromonas translucida* strains from fish and sediments exhibited combinations of *VAN*, *SULF*, *qacB*, *oqxB* and, in some isolates, *qnrA*. Comparable ARG patterns have been documented in Antarctic wildlife. Sulfonamide and quinolone resistance genes have been detected in birds, seal faeces and marine sediments [50], while vancomycin resistance genes have been reported in penguins and leopard seals [70]. Genes conferring resistance to beta-lactams, tetracyclines and aminoglycosides—including *blaTEM*, *tetA* and *aac(3)-II*, have also been reported in Adélie penguins and South polar skuas [71]. Together with findings in penguins and Antarctic fur seals [3], these data underscore the broad distribution of ARGs across Antarctic fauna.

Beyond fish tissues, our results show that surface seawater and water column isolates also carried multiple ARGs. *Psychrobacter* spp. from seawater harboured *TET*, *VAN*, *SULF*, *qacB* and *oqxB*, mirroring the profiles found in fish-associated isolates. *Metaplanococcus flavidum* strains from different water layers carried subsets of these genes, including *SULF*, *VAN*, *qacB* and *oqxB*, indicating heterogeneous but persistent ARG circulation across the water column. These findings align with previous evidence showing that Antarctic matrices—including soils, sediments and wildlife—harbour diverse resistance genes despite the continent’s remoteness. Indigenous soil bacteria carry ARGs such as *tet(B)*, *bla_PER-2*, *strA* and *strB* [72], while multidrug-resistant *Pseudomonas* strains resistant to ten or more antibiotics have been isolated from pristine areas [73]. Our data extend this concept to coastal marine microbiota and fish-associated bacteria, indicating that ARGs are not restricted to terrestrial environments. A key concern is the potential for horizontal gene transfer (HGT). Several Antarctic Acinetobacter and *Pseudomonas* strains carry mobile genetic elements associated with ARG dissemination [49], and our detection of multi-resistant isolates harbouring both quinolone (*qnrA*, *oqxB*) and disinfectant-resistance genes (*qacB*) reinforces this possibility. While HGT cannot be confirmed without targeted genomic analyses, the presence of complex ARG combinations in genera commonly associated with plasmids and integrons suggests that gene exchange may occur in these communities. Anthropogenic inputs remain an important driver of ARG dissemination in Antarctica, particularly near research stations. Wastewater discharge, human occupation and wildlife acting as biological vectors have been implicated in spreading resistant bacteria [74]. Studies across the Fildes Peninsula have reported bacteria resistant to ciprofloxacin and sulfamethazine in soils, lakes, marine sediments and faeces [47].

Our detection of ARGs across fish, seawater and sediments in Terra Nova Bay is consistent with a scenario where both natural and human-related pressures contribute to shaping the coastal resistome. Finally, the integration of ARG-positive bacteria into Antarctic food webs warrants attention. *T. bernacchii* is a key ecologically and trophically relevant species, and the presence of multidrug-resistant strains within its microbiota raises questions about the persistence, transfer and ecological consequences of ARGs across trophic levels. Previous work has warned that antimicrobial resistance in remote ecosystems may alter microbial community dynamics and potentially affect higher trophic levels [75]. Our findings support this concern and highlight the need for broader One Health-oriented surveillance of ARGs in Antarctic marine systems.

## 5. Conclusions

This study demonstrates that heterotrophic marine bacteria isolated from *Trematomus bernacchii*, seawater, and marine sediments in the Antarctic marine environment exhibit widespread multidrug resistance and high tolerance to heavy metals.

Taxonomic analysis revealed that resistant phenotypes were associated with bacteria belonging to the phyla Pseudomonadota and Bacillota, including species of *Pseudomonas*, *Pseudoalteromonas*, *Psychrobacter*, and *Metaplanococcus*. Screening for antibiotic resistance genes confirmed the presence of multiple ARGs, including VAN, TET, SULF, qacB, qnrA, and oqxB, across isolates from different environmental matrices, supporting the phenotypic resistance patterns observed. Overall, these findings provide clear evidence that Antarctic marine bacteria, including free-living and host-associated strains, possess complex and diversified resistance profiles to both antibiotics and heavy metals.

The consistent co-occurrence of antibiotic and metal resistance suggests that environmental selective pressures may play a role in maintaining these traits, even in remote polar ecosystems. Our findings reveal a widespread presence of ARB and HMRB, emphasizing the significant impact of both natural environmental pressures and human activities, including wastewater discharge and localized contamination near research stations.

The presence of ARBs in the marine environment of Antarctica, particularly in fish species such as *T. bernacchii*, raises ecological and public health concerns resulting from the potential for dissemination of resistance genes through trophic interactions and broader microbial networks. The results underscore the need for continuous monitoring of resistance determinants and improved wastewater management practices to mitigate the risks posed by antimicrobial and heavy metal contaminants in Antarctica.

## Figures and Tables

**Table 1:** Genetic assays and bacterial identification of the 12 selected strains. *TET*: tetracycline, *VAN*: vancomycin; *SULF*: sulfonamides; *qacB* quaternary ammonium compounds; *qnrA*: quinolones; *oqxB*: oxazolidinones and quinolones.

Source	Strain n.	Bacterial Genus	*TET*	*VAN*	*SULF*	*qacB*	*qnrA*	*oqxB*
*Trematomus* *bernacchii*	RB67	*Pseudomonas* *Versuta*	−	+	−	+	+	+
*Trematomus* *bernacchii*	RB68	*Pseudomonas* *versuta*	−	+	−	+	+	+
*Trematomus* *bernacchii*	RB139	*Pseudoalteromonas translucida*	−	+	+	+	+	+
*Trematomus bernacchii*	RB161	*Pseudoalteromonas translucida*	−	+	+	+	−	+
*Trematomus bernacchii*	RB170	*Pseudoalteromonas translucida*	−	+	−	+	+	+
*Trematomus* *bernacchii*	RB206	*Psychrobacter* sp.	−	+	+	−	+	+
*Trematomus* *bernacchii*	TB341	*Psychrobacter* sp.	−	+	−	+	−	+
Surface water	RB4	*Psychrobacter* sp.	+	+	+	+	−	+
Surface water	RB5	*Metaplanococcus* *flavidum*	−	+	−	−	−	+
Column Water	RB52	*Metaplanococcus* *Flavidum*	−	+	−	+	−	+
Column Water	RB242	*Psychrobacter* sp.	+	+	+	+	−	+
Sediment	TB188	*Pseudoalteromonas translucida*	+	−	−	+	+	+

## Data Availability

Original data are available as Supplementary Materials.

## Supplementary Materials

The following supporting information can be downloaded at https://www.mdpi.com/article/10.3390/ani16010051/s1, Table S1. Origin of the bacterial strains and relative environmental data, A-Road Bay site, B-Thetys Bay site. Table S2. Antibiotics tested in this study, acronym and concentrations. Table S3. Genetic assays of the marine heterotrophic bacterial strains. *TET*: tetracycline, *VAN*: vancomycin; *SULF*: sulfonamides; *qacB* quaternary ammonium compounds; *qnrA*: quinolones; *oqxB*: oxazolidinones and quinolones; ng: no growth. Table S4. Primers used in this study (Eurofins, genomic Italy srl). Table S5. Heavy metal resistance for the 12 identified bacterial strains. x: resistant, 0: sensitive.
