# Supplementary material for: Antibiotic and Heavy Metal Resistance in Marine Bacteria from Terra Nova Bay (Ross Sea): Insights from Wild Fish and Environmental Samples
Source: Animals (Basel). 2025 Dec 24;16(1):51. doi: 10.3390/ani16010051 (PMC12784928; doi:10.3390/ani16010051)
Supplement: Supplementary file 1 [file animals-16-00051-s001.zip › animals-4043778-supplementary.docx]

**Supplementary Materials:**

Antibiotic and Heavy Metal Resistance in Marine Bacteria from Terra Nova Bay (Ross Sea): Insights from Wild Fish and Environmental Samples

Enrico Gugliandolo ^1^, Bilal Mghili ^2^, Francesca Fabrizi ^3,4^, Kannan Gunasekaran ^5^, Francesco Smedile ^6^, Francesca Inferrera ^7^, Sabrina Natale ^7^, Teresa Romeo ^8,9,10,11^, Erika Arcadi ^8^, Syed Sikandar Habib ^12^, Maurizio Azzaro ^6^, Teresa Bottari ^3,11,^* and Monique Mancuso ^3,11^

^1^ Department of Veterinary Science, University of Messina, 98168 Messina, Italy;
enrico.gugliandolo@unime.it

^2^ LESCB, URL-CNRST N°18, Faculty of Sciences, Abdelmalek Essaadi University, Tetouan 93000, Morocco; b.mghili@uae.ac.ma

^3^ Institute for Marine Biological Resources and Biotechnology (IRBIM), National Research Council (CNR), 98122 Messina, Italy; francescafabrizi@cnr.it (F.F.); monique.mancuso@cnr.it (M.M.)

^4^ Scuola Universitaria Superiore IUSS Pavia, 27100 Pavia, Italy

^5^ Department of Marine Science, Faculty of Science, Chulalongkorn University, Bangkok 10330, Thailand; bk.guna18@gmail.com

^6^ Institute of Polar Sciences (ISP), National Research Council (CNR), 98122 Messina, Italy; francesco.smedile@cnr.it (F.S.); maurizio.azzaro@cnr.it (M.A.)

^7^ Department of Chemical, Biological, Pharmaceutical and Environmental Sciences, University of Messina, 98166 Messina, Italy; francesca.inferrera@studenti.unime.it (F.I.); sabrina.natale@unime.it (S.N.)

^8^ Stazione Zoologica “Anton Dohrn”, Sicily Marine Centre, 98167 Messina, Italy; teresa.romeo@szn.it (T.R.); erika.arcadi@szn.it (E.A.)

^9^ Stazione Zoologica Anton Dohrn, Sicily Marine Centre, Department of Biology and Evolution of Marine Organisms (BEOM), Via Dei Mille 46, 98057 Milazzo, Italy

^10^ National Institute for Environmental Protection and Research, 98057 Milazzo, Italy

^11^ National Biodiversity Future Center (NBFC), 90133 Palermo, Italy

^12^ Department of Zoology, University of Sargodha, Sargodha Punjab 40100, Pakistan; sikandarzoo00@yahoo.com

***** Correspondence: teresa.bottari@cnr.it

**Table S1.** Origin of the bacterial strains and relative environmental data, A-Road Bay site, B- Thetys Bay site.

| Source | Strain n. | | | Location | Date | Depth (m) | pH | Oxygen (mg/l) | Salinity (PSU) | Temperature (°C) |
| --- | --- | --- | --- | --- | --- | --- | --- | --- | --- | --- |
| *Trematomus bernacchii* | | | RB67 | A | 20/11/21 | 16 | 8.00 | 10.72 | 33.75 | -1.94 |
| *Trematomus bernacchii* | | | RB68 | A | 20/11/21 | 16 | 8.00 | 10.72 | 33.75 | -1.94 |
| *Trematomus bernacchii* | | RB71 | | A | 20/11/21 | 16 | 8.00 | 10.72 | 33.75 | -1.94 |
| *Trematomus bernacchii* | | RB73 | | A | 20/11/21 | 16 | 8.00 | 10.72 | 33.75 | -1.94 |
| *Trematomus bernacchii* | | RB79 | | A | 20/11/21 | 16 | 8.00 | 10.72 | 33.75 | -1.94 |
| *Trematomus bernacchii* | | RB88 | | A | 20/11/21 | 16 | 8.00 | 10.72 | 33.75 | -1.94 |
| *Trematomus bernacchii* | | RB90 | | A | 20/11/21 | 16 | 8.00 | 10.72 | 33.75 | -1.94 |
| *Trematomus bernacchii* | | RB139 | | A | 20/11/21 | 16 | 8.00 | 10.72 | 33.75 | -1.94 |
| *Trematomus bernacchii* | | RB161 | | A | 20/11/21 | 16 | 8.00 | 10.72 | 33.75 | -1.94 |
| *Trematomus bernacchii* | | RB162 | | A | 20/11/21 | 16 | 8.00 | 10.72 | 33.75 | -1.94 |
| *Trematomus bernacchii* | | RB163 | | A | 20/11/21 | 16 | 8.00 | 10.72 | 33.75 | -1.94 |
| *Trematomus bernacchii* | | RB164 | | A | 20/11/21 | 16 | 8.00 | 10.72 | 33.75 | -1.94 |
| *Trematomus bernacchii* | | RB165 | | A | 20/11/21 | 16 | 8.00 | 10.72 | 33.75 | -1.94 |
| *Trematomus bernacchii* | | RB166 | | A | 20/11/21 | 16 | 8.00 | 10.72 | 33.75 | -1.94 |
| *Trematomus bernacchii* | | RB167 | | A | 20/11/21 | 16 | 8.00 | 10.72 | 33.75 | -1.94 |
| *Trematomus bernacchii* | | RB170 | | A | 20/11/21 | 16 | 8.00 | 10.72 | 33.75 | -1.94 |
| *Trematomus bernacchii* | | RB171 | | A | 20/11/21 | 16 | 8.00 | 10.72 | 33.75 | -1.94 |
| *Trematomus bernacchii* | | RB173 | | A | 20/11/21 | 16 | 8.00 | 10.72 | 33.75 | -1.94 |
| *Trematomus bernacchii* | | RB206 | | A | 20/11/21 | 16 | 8.00 | 10.72 | 33.75 | -1.94 |
| *Trematomus bernacchii* | | RB 285 | | A | 21/11/21 | 16 | 8.00 | 10.72 | 33.75 | -1.94 |
| *Trematomus bernacchii* | | TB341 | | B | 21/11/21 | 16 | 7.98 | 9.20 | 33.78 | -2.00 |
| *Trematomus bernacchii* | | RB349 | | A | 24/11/21 | 16 | 8.00 | 10.72 | 33.75 | -1.94 |
| Superficial water | | RB1 | | A | 03/11/21 | 0 | 7.72 | 8.41 | 33.79 | -1.98 |
| Superficial water | | RB2 | | A | 03/11/21 | 0 | 7.72 | 8.41 | 33.79 | -1.98 |
| Superficial water | | RB4 | | A | 03/11/21 | 0 | 7.72 | 8.41 | 33.79 | -1.98 |
| Superficial water | | RB5 | | A | 03/11/21 | 0 | 7.72 | 8.41 | 33.79 | -1.98 |
| Superficial water | | RB6 | | A | 03/11/21 | 0 | 7.72 | 8.41 | 33.79 | -1.98 |
| Superficial water | | RB15 | | A | 03/11/21 | 0 | 7.72 | 8.41 | 33.79 | -1.98 |
| Superficial water | | RB16 | | A | 03/11/21 | 0 | 7.72 | 8.41 | 33.79 | -1.98 |
| Superficial water | | RB17 | | A | 03/11/21 | 0 | 7.72 | 8.41 | 33.79 | -1.98 |
| Superficial water | | RB32 | | A | 03/11/21 | 0 | 7.72 | 8.41 | 33.79 | -1.98 |
| Superficial water | | RB38 | | A | 03/11/21 | 0 | 7.72 | 8.41 | 33.79 | -1.98 |
| Superficial water | | RB41 | | A | 03/11/21 | 0 | 7.72 | 8.41 | 33.79 | -1.98 |
| Superficial water | | RB45 | | A | 03/11/21 | 0 | 7.72 | 8.41 | 33.79 | -1.98 |
| Column Water | | RB50 | | A | 03/11/21 | 10 | 7.82 | 9.44 | 33.78 | -1.99 |
| Column Water | | RB51 | | A | 03/11/21 | 10 | 7.82 | 9.44 | 33.78 | -1.99 |
| Column Water | | RB52 | | A | 03/11/21 | 10 | 7.82 | 9.44 | 33.78 | -1.99 |
| Column Water | | RB54 | | A | 03/11/21 | 10 | 7.82 | 9.44 | 33.78 | -1.99 |
| Column Water | | RB57 | | A | 03/11/21 | 10 | 7.82 | 9.44 | 33.78 | -1.99 |
| Column Water | | RB242 | | A | 21/11/21 | 16 | 8.00 | 10.72 | 33.75 | -1.94 |
| Column Water | | RB243 | | A | 21/11/21 | 16 | 8.00 | 10.72 | 33.75 | -1.94 |
| Column Water | | RB434 | | A | 03/12/21 | 10 | 8.10 | 8.64 | 33.74 | -1.89 |
| Column Water | | RB524 | | A | 11/12/21 | 10 | 8.31 | 11.66 | 33.65 | -1.33 |
| Column Water | | TB539 | | B | 11/12/21 | 16 | 8.28 | 7.96 | 33.64 | -1.28 |
| Column Water | | TB549 | | B | 11/12/21 | 16 | 8.28 | 7.96 | 33.64 | -1.28 |
| Bottom water | | RB536 | | A | 11/12/21 | 16 | 8.28 | 10.04 | 33.51 | -1.19 |
| Bottom water | | RB537 | | A | 11/12/21 | 16 | 8.28 | 10.04 | 33.51 | -1.19 |
| Sediment | | TB188 | | B | 20/11/21 | 16 | 7.98 | 9.20 | 33.78 | -2.00 |
| Sediment | | TB189 | | B | 20/11/21 | 16 | 7.98 | 9.20 | 33.78 | -2.00 |
| Sediment | | TB385 | | B | 30/11/21 | 16 | 8.09 | 8.79 | 33.7 | -1.82 |

**Table S2.** Antibiotics tested in this study, acronym and concentrations.

| Antibiotics | Acronym | Concentration |
| --- | --- | --- |
| Amoxicillin | AML | 10 µg |
| Amoxicillin + Clavulanic Acid | AMC | 3 µg |
| Azithromycin | AZM | 15 µg |
| Cefotaxime | CTX | 30 µg |
| Cephalexin | CL | 30 µg |
| Cephoxitin | FOX | 30 µg |
| Chloramphenicol | C | 30 µg |
| Ciprofloxacin | CIP | 5 µg |
| Clindamycin | CD | 30 µg |
| Erythromycin | E | 15 µg |
| Flumequine | UB | 30 µg |
| Fosfomycin | FOS | 50 µg |
| Gentamycin | CN | 30 µg |
| Kanamycin | K | 30 µg |
| Levofloxacin | LEV | 5 µg |
| Neomycin | N | 30 µg |
| Nitrofurantoin | F | 200 µg |
| Novobiocin | NV | 5 µg |
| Oxacillin | OX | 5 µg |
| Penicillin G | P | 10 µg |
| Piperacillin | PRL | 30 µg |
| Polymyxin B | PB | 300 µg |
| Rifampicin | RD | 30 µg |
| Rifampicin | RA | 5 µg |
| Tetracycline | TE | 30 µg |
| Tobramycin | TOB | 10 µg |
| Trimethoprim/Sulphathiazole | SSXT | 25 µg |
| Vancomycin | VA | 30 µg |

**Table S3.** Genetic assays of the marine heterotrophic bacterial strains. *TET*: tetracycline, *VAN*: vancomycin; *SULF*: sulfonamides; *qacB* quaternary ammonium compounds; *qnrA*: quinolones; *oqxB*: oxazolidinones and quinolones; ng: no growth.

| Bacterial strains | *TET* | *VAN* | *SULF* | *qacB* | *qnrA* | *oqxB* |
| --- | --- | --- | --- | --- | --- | --- |
| RB1 | - | + | + | - | - | + |
| RB2 | + | + | + | - | - | - |
| RB4 | + | + | + | + | - | + |
| RB5 | - | + | - | - | - | + |
| RB6 | - | + | + | - | - | + |
| RB15 | - | + | - | - | - | - |
| RB16 | + | + | - | - | - | - |
| RB17 | - | + | - | - | - | - |
| RB32 | - | + | - | - | - | + |
| RB41 | - | + | - | - | - | - |
| RB45 | - | - | + | - | - | - |
| RB48 | - | + | - | - | - | - |
| RB50 | - | - | - | - | - | + |
| RB51 | - | + | - | + | - | - |
| RB52 | - | + | - | + | - | + |
| RB67 | - | + | - | + | + | + |
| RB68 | - | + | - | + | + | + |
| RB70 | - | - | - | + | - | + |
| RB71 | - | + | - | - | + | + |
| RB73 | - | + | - | + | + | + |
| RB74 | - | + | - | + | + | - |
| RB79 | - | + | - | + | - | + |
| RB88 | ng | Ng | ng | Ng | ng | ng |
| RB90 | ng | Ng | ng | Ng | ng | ng |
| RB139 | - | + | + | + | + | + |
| RB161 | - | + | + | + | - | + |
| RB162 | ng | Ng | ng | Ng | ng | ng |
| RB163 | - | + | + | + | + | - |
| RB164 | - | + | + | + | - | - |
| RB165 | - | + | - | + | - | - |
| RB166 | - | + | + | - | - | - |
| RB167 | - | + | - | - | - | + |
| RB170 | - | + | - | + | + | + |
| RB171 | - | + | + | + | - | + |
| RB173 | - | + | + | + | - | + |
| TB188 | + | - | - | + | + | + |
| TB189 | + | - | - | + | + | - |
| RB206 | - | + | + | - | + | + |
| RB242 | + | + | + | + | - | + |
| RB243 | - | + | - | - | - | + |
| TB341 | - | + | - | + | - | + |
| RB349 | + | - | - | - | - | - |
| TB385 | - | + | - | - | - | - |
| RB524 | - | + | - | - | - | - |
| RB536 | - | - | + | - | - | - |
| RB537 | - | - | - | - | - | - |
| TB538 | - | + | - | - | - | - |
| TB549 | - | - | - | - | - | - |

**Table S4.** Primers used in this study (Eurofins, genomic Italy srl).

| Gene | Forward | Reverse | Tm (°C) |
| --- | --- | --- | --- |
| *tetM* | ATCCTTTCTGGGCTTCCATT | TCCGTCACATTCCAACCATA | 55.3 |
| *vanC* | GGGAAGATGGCAGTATCCAAGG | GCTTGATGCAGCAGCCATTT | 62.1 |
| *sul3* | GGTTGAAGATGGAGCAGATG | GCCTTAATGACAGGTTTGAGTC | 58.4 |
| *quacB* | CACAATGGTTACAGGTTGTGG | AATGGCTGCAGTTCCAATTC | 57.9 |
| *qnrA* | AGGATTTCTCACGCCAGGATT | CCGCTTTCAATGAAACTGCA | 57.9 |
| *Oqxb* | TCCTGATCTCCATTAACGCCCA | ACCGGAACCCATCTCGATGC | 61.4 |

**Table S5.** Heavy metal resistance for the 12 identified bacterial strains. x: resistant, 0: sensitive.

|  |  | Heavy metals concentrations (ppm) | | | | | |
| --- | --- | --- | --- | --- | --- | --- | --- |
| Strain n. | Genus | 10 | | 100 | 1000 | 5000 | 10000 |
|  | Pb(C2H3O2) | |  |  |  |  |  |
| RB67 | *Pseudomonas versuta* | | x | x | x | x | X |
| RB68 | *Pseudomonas versuta* | | x | x | x | x | X |
| RB139 | *Pseudoalteromonas*  *translucida* | | x | x | x | x | X |
| RB161 | *Pseudoalteromonas*  *translucida* | | x | x | x | x | X |
| RB188 | *Pseudoalteromonas*  *translucida* | | x | x | x | x | x |
| RB206 | *Psychrobacter sp.* | | x | x | x | x | x |
| TB341 | *Psychrobacter sp.* | | x | x | x | x | x |
| RB4 | *Psychrobacter sp.* | | x | x | x | x | x |
| RB5 | *Metaplanococcus flavidum* | | x | x | x | x | x |
| RB170 | *Pseudoalteromonas*  *translucida* | | x | x | x | x | x |
| RB52 | *Metaplanococcus flavidum* | | x | x | x | x | x |
| RB242 | *Psychrobacter sp.* | | x | x | x | x | x |
|  | 3CdSO₄·8H₂O | |  |  |  |  |  |
| RB67 | *Pseudomonas versuta* | | x | x | x | x | x |
| RB68 | *Pseudomonas versuta* | | x | x | x | x | x |
| RB139 | *Pseudoalteromonas*  *translucida* | | x | x | x | x | x |
| RB161 | *Pseudoalteromonas*  *translucida* | | x | x | x | x | x |
| RB188 | *Pseudoalteromonas*  *translucida* | | x | x | x | x | x |
| RB206 | *Psychrobacter sp.* | | x | x | x | x | x |
| TB341 | *Psychrobacter sp.* | | x | x | x | x | x |
| RB4 | *Psychrobacter sp.* | | x | x | x | x | x |
| RB5 | *Metaplanococcus flavidum* | | x | x | x | x | x |
| RB170 | *Pseudoalteromonas*  *translucida* | | x | x | x | x | x |
| RB52 | *Metaplanococcus flavidum* | | x | x | x | x | x |
| RB242 | *Psychrobacter sp.* | | x | x | x | x | x |
|  | NiCl2 | |  |  |  |  |  |
| RB67 | *Pseudomonas versuta* | | x | x | x | x | x |
| RB68 | *Pseudomonas versuta* | | x | x | x | x | x |
| RB139 | *Pseudoalteromonas*  *translucida* | | x | x | x | x | x |
| RB161 | *Pseudoalteromonas*  *translucida* | | x | x | x | x | x |
| RB188 | *Pseudoalteromonas*  *translucida* | | x | x | x | x | x |
| RB206 | *Psychrobacter sp.* | | x | x | x | x | x |
| TB341 | *Psychrobacter sp.* | | x | x | x | x | x |
| RB4 | *Psychrobacter sp.* | | x | x | x | 0 | 0 |
| RB5 | *Metaplanococcus flavidum* | | x | x | 0 | 0 | 0 |
| RB170 | *Pseudoalteromonas*  *translucida* | | x | x | x | x | x |
| RB52 | *Metaplanococcus flavidum* | | x | x | x | x | x |
| RB242 | *Psychrobacter sp.* | | x | x | x | x | x |
|  | CoCl2 | |  |  |  |  |  |
| RB67 | *Pseudomonas versuta* | | x | x | x | x | x |
| RB68 | *Pseudomonas versuta* | | x | x | x | x | x |
| RB139 | *Pseudoalteromonas*  *translucida* | | x | x | x | x | x |
| RB161 | *Pseudoalteromonas*  *translucida* | | x | x | x | x | x |
| RB188 | *Pseudoalteromonas*  *translucida* | | x | x | x | x | x |
| RB206 | *Psychrobacter sp.* | | x | x | x | x | x |
| TB341 | *Psychrobacter sp.* | | x | x | x | x | x |
| RB4 | *Psychrobacter sp.* | | x | x | x | x | x |
| RB5 | *Metaplanococcus flavidum* | | x | x | x | x | 0 |
| RB170 | *Pseudoalteromonas*  *translucida* | | x | x | x | x | x |
| RB52 | *Metaplanococcus flavidum* | | x | x | x | x | x |
| RB242 | *Psychrobacter sp.* | | x | x | x | x | x |
|  | CuSO4 | |  |  |  |  |  |
| RB67 | *Pseudomonas versuta* | | x | x | x | x | x |
| RB68 | *Pseudomonas versuta* | | x | x | x | x | x |
| RB139 | *Pseudoalteromonas*  *translucida* | | x | x | x | x | x |
| RB161 | *Pseudoalteromonas*  *translucida* | | x | x | x | x | x |
| RB188 | *Pseudoalteromonas*  *translucida* | | x | x | x | x | x |
| RB206 | *Psychrobacter sp.* | | x | x | x | x | x |
| TB341 | *Psychrobacter sp.* | | x | x | x | x | x |
| RB4 | *Psychrobacter sp.* | | x | x | x | x | x |
| RB5 | *Metaplanococcus flavidum* | | x | x | x | x | x |
| RB170 | *Pseudoalteromonas*  *translucida* | | x | x | x | x | x |
| RB52 | *Metaplanococcus flavidum* | | x | x | x | x | x |
| RB242 | *Psychrobacter sp.* | | x | x | x | x | x |
|  | HgCl2 | |  |  |  |  |  |
| RB67 | *Pseudomonas versuta* | | x | x | 0 | 0 | 0 |
| RB68 | *Pseudomonas versuta* | | x | x | 0 | 0 | 0 |
| RB139 | *Pseudoalteromonas*  *translucida* | | x | x | x | 0 | 0 |
| RB161 | *Pseudoalteromonas*  *translucida* | | x | x | x | 0 | 0 |
| RB188 | *Pseudoalteromonas*  *translucida* | | x | x | x | x | 0 |
| RB206 | *Psychrobacter sp.* | | x | x | 0 | 0 | 0 |
| TB341 | *Psychrobacter sp.* | | x | x | x | 0 | 0 |
| RB4 | *Psychrobacter sp.* | | 0 | 0 | 0 | 0 | 0 |
| RB5 | *Metaplanococcus flavidum* | | x | x | x | 0 | 0 |
| RB170 | *Pseudoalteromonas*  *translucida* | | x | x | 0 | 0 | 0 |
| RB52 | *Metaplanococcus flavidum* | | x | x | 0 | 0 | 0 |
| RB242 | *Psychrobacter sp.* | | x | x | x | 0 | 0 |
|  | ZnSO₄* 7 H₂O | |  |  |  |  |  |
| RB67 | *Pseudomonas versuta* | | x | x | x | x | x |
| RB68 | *Pseudomonas versuta* | | x | x | x | x | x |
| RB139 | *Pseudoalteromonas*  *translucida* | | x | x | x | x | x |
| RB161 | *Pseudoalteromonas*  *translucida* | | x | x | x | x | x |
| RB188 | *Pseudoalteromonas*  *translucida* | | x | x | x | x | x |
| RB206 | *Psychrobacter sp.* | | x | x | x | x | x |
| TB341 | *Psychrobacter sp.* | | x | x | x | x | x |
| RB4 | *Psychrobacter sp.* | | x | x | x | x | 0 |
| RB5 | *Metaplanococcus flavidum* | | x | x | x | x | x |
| RB170 | *Pseudoalteromonas*  *translucida* | | x | x | x | x | x |
| RB52 | *Metaplanococcus flavidum* | | x | x | x | x | x |
| RB242 | *Psychrobacter sp.* | | x | x | x | x | x |
